# Development and Validation of a Gene-Based Model for Outcome Prediction in Germ Cell Tumors Using a Combined Genomic and Expression Profiling Approach

**DOI:** 10.1371/journal.pone.0142846

**Published:** 2015-12-01

**Authors:** James E. Korkola, Sandy Heck, Adam B. Olshen, Darren R. Feldman, Victor E. Reuter, Jane Houldsworth, George J. Bosl, R. S. K. Chaganti

**Affiliations:** 1 Cell Biology Program, Sloan-Kettering Institute for Cancer Research, New York, New York, United States of America; 2 Departments of Medicine and Pathology, Weill Cornell Medical College, New York, New York, United States of America; 3 Department of Epidemiology and Biostatistics, Memorial Sloan Kettering Cancer Center, New York, New York, United States of America; 4 Department of Medicine, Memorial Sloan-Kettering Cancer Center, New York, New York, United States of America; 5 Department of Pathology, Memorial Sloan-Kettering Cancer Center, New York, New York, United States of America; University of Maryland, UNITED STATES

## Abstract

Germ Cell Tumors (GCT) have a high cure rate, but we currently lack the ability to accurately identify the small subset of patients who will die from their disease. We used a combined genomic and expression profiling approach to identify genomic regions and underlying genes that are predictive of outcome in GCT patients. We performed array-based comparative genomic hybridization (CGH) on 53 non-seminomatous GCTs (NSGCTs) treated with cisplatin based chemotherapy and defined altered genomic regions using Circular Binary Segmentation. We identified 14 regions associated with two year disease-free survival (2yDFS) and 16 regions associated with five year disease-specific survival (5yDSS). From corresponding expression data, we identified 101 probe sets that showed significant changes in expression. We built several models based on these differentially expressed genes, then tested them in an independent validation set of 54 NSGCTs. These predictive models correctly classified outcome in 64–79.6% of patients in the validation set, depending on the endpoint utilized. Survival analysis demonstrated a significant separation of patients with good versus poor predicted outcome when using a combined gene set model. Multivariate analysis using clinical risk classification with the combined gene model indicated that they were independent prognostic markers. This novel set of predictive genes from altered genomic regions is almost entirely independent of our previously identified set of predictive genes for patients with NSGCTs. These genes may aid in the identification of the small subset of patients who are at high risk of poor outcome.

## Background

Germ cell tumors (GCTs) are the most common solid malignancy in young adult men, with a peak incidence between the ages of 18 and 35 [1]. Fortunately, the cure rate for this disease is very high, such that greater than 90% of all new patients achieve long-term survival [1]. Even when patients present with metastatic disease, cures can be achieved in nearly 80% of cases [1].

Currently, patients are risk stratified using the International Germ Cell Cancer Collaborative Group (IGCCCG) prognostic classification, which is based on histology (seminoma [SEM] versus non-seminoma [NSGCT]), serum marker levels of alpha-fetoprotein (AFP), human chorionic gonadatropin (HCG), and lactate dehydrogenase (LDH), the presence of non-pulmonary visceral metastases, and the site of the primary tumor (gonadal versus mediastinal) [1]. Based on these parameters, patients are assigned to good, intermediate, and poor risk categories. Treatment decisions are based on this risk stratification, with patients in the good risk category typically receiving either three cycles of BEP (bleomycin, etoposide, and cisplatin) or four cycles of EP (etoposide and cisplatin) [1, 2], while intermediate and poor risk patients receive four cycles of BEP [1, 3]. While the IGCCCG classification is useful for making treatment decisions, it does not perform as well in predicting patient outcome. For example, approximately 45% of poor risk patients are cured [1], but we currently have no means of distinguishing those who will die of disease from those who will be cured. The addition of prognostic molecular markers could improve patient outcome prediction, as well as identify patients who might benefit from more aggressive or alternative treatments that are traditionally reserved for second line or salvage therapies, such as high dose chemotherapy with stem cell rescue or ifosfamide based therapies [4].

To this end, we previously performed a large expression based study to identify genes associated with patient outcome [5]. While we were successful in developing gene based models that were highly predictive of outcome in NSGCT patients, this approach does have shortcomings. Specifically, since the model building methods we utilized are based on average expression in the poor versus good outcome groups, genes that may be highly predictive of outcome but are expressed in small subsets of tumors will have limited predictive power in the whole panel and thus be excluded. Recently, computational analyses have been described to identify these outlier populations [6], but we chose an alternative approach making use of the genomic copy number analyses. We examined genomic alterations for correlation with patient outcome. We identified a number of regions, some of which were altered infrequently, that had very strong correlations with outcome. Since we lacked an independent CGH data set, we instead examined expression of genes that map to the altered regions associated with outcome to identify potential predictive genes and build predictive models. We then validated these predictive gene expression models in an independent tumor cohort to determine their clinical utility.

## Methods

### Tumor Material

This study, including genomic and expression profiling as well as correlation of results with patient demographics, clinical characteristics, and outcome was approved by the institutional review board (IRB) at Memorial Sloan Kettering Cancer (WA0069-06). Available fresh frozen tumor tissue from patients with advanced GCT who had previously provided IRB-approved written informed consent to have their tumor used for research purposes was identified and obtained through query of our Pathology Department tumor bank. We have previously reported both the genomic profiling and expression profiling of these tumors [7–9]. The data for these specimens has been deposited in the GEO database (genomic profiles available under accession number GSE8614; expression profiles are available under accession numbers GSE3218 and GSE10783). Of the 74 tumors we previously profiled by array CGH, 53 were NSGCT that had been treated with cisplatin-based chemotherapy and had patient follow-up data, which were utilized for this study. The validation set consisted of an additional 54 NSGCT specimens for which we had no array CGH data but had expression data from our previous expression profiling studies. Of these 54, 49 had sufficient follow-up for 5yDSS calculations. The clinical information for all samples used in this study and our previous expression based study [5] are included as S1 Table. Normal testes samples from aged matched patients were used as normal controls, as described previously [8].

### DNA Isolation and array CGH

DNA was isolated as previously described [7]. Array CGH (aCGH) was performed as previously described using 1 Mb resolution BAC chips (Spectral Genomics) [7]. Additional copy number analysis was done using high-resolution Agilent 44K snp chips, and was performed by the Integrated Genomic Operation at MSKCC.

### Statistical Analysis

The processing of the data was described previously [7, 8]. Briefly, for aCGH analysis, ratio data (test/reference channel) were generated using Genepix Pro, normalized, and averaged. After removal of polymorphic, unmapped, or absent clones, the circular binary segmentation (CBS) algorithm was used to identify segmental gains and losses along autosomes as previously described by us [7, 10]. Fisher’s exact test was used to test for associations between copy number and patient outcome. We looked at two different endpoints: i) two year disease free survival [2yDFS], which divided patients into good outcome (no evidence of disease two years after the start of their treatment) or poor outcome (never disease free or recurrence of disease within two years after the start of treatment); and ii) five year disease specific survival [5yDSS]), which divided patients into good outcome (still alive five years after initial treatment for disease) and poor outcome (dead of disease within five years of initial treatment for disease). Genomic regions were defined based on: i) statistically significant associations of the BAC clone with outcome; and ii) proximity to the next significant BAC clone. For the proximity cutoff, clones had to be within 11.5 Mb of the next significant clone. This conservative cutoff was chosen to account for instances where there were missing data, excluded data, or large gaps between adjacent BAC clones (such as those spanning centromeric or heterochromatic regions). In practice, the gap between adjacent BACs was much smaller. There were only two pairs of clones that were close to the 11.5 Mb cutoff (RP11-24G16 and RP11-89N6 on chr4, 10.4 Mb apart, associated with 5yDSS; and RP11-91M2 and RP11-62C3 on chr9, 11.4 Mb apart, associated with 2yDFS). The median gap was 0.9 Mb and the mean gap was 1.2 Mb for adjacent clones defining the altered region. All regions consisted of at least two clones, with the exception of a single BAC clone (RP11-404D6) on chr17 that was associated with 2yDFS. Potential target genes mapping to altered genomic regions that showed significant associations with outcome were identified by testing for differential gene expression between samples that had the alteration compared to samples that had normal copy number using Significance Analysis for Microarrays (SAM) [11]. The analysis was performed to choose the first point where the False Discovery Rate (FDR) dropped below 10%. A particular emphasis was placed on significant genes that showed at least a two-fold change in expression relative to tumors with normal copy number or those that showed at least a three-fold change in expression relative to normal testes specimens. All significant genes that met these criteria were used to build a model using Prediction Analysis for Microarrays (PAM) [12]. This predictive gene set was then tested in the independent tumor cohort for which we had expression and follow-up data but no genomic copy number data. This independent cohort consisted of two tumor sets that were profiled at different times. To avoid differences that may have arisen as a result of using different batches of arrays, the second set of tumors was normalized so that the median expression value for each gene matched the expression value for the corresponding gene from the first set of tumors (which were a subset of the tumors used to build the predictive model but which lacked copy number data).

## Results

We previously completed a large study examining copy number changes in tumors from 74 GCT patients [7]. In the present study, we tested for associations between copy number alterations and outcome in a subset of 53 of NSGCT patients that were treated with cisplatin based chemotherapy and for whom we had clinical outcome information. Although these tumors represent a diverse set of NSGCT specimens with multiple histologic subtypes, which we have previously described [9], histology has not been shown as being an important predictive or prognostic marker for NSGCT, as evidenced by its absence in the IGCCCG risk stratification [13]. Thus, we used the entire cohort to examine two different outcome endpoints (2y Disease Free Survival (DFS) and 5y Disease Specific Survival (DSS)) using Fisher’s exact test for this study.

### 2yDFS Associations

We identified 14 regions (5 gains, 9 losses) that were associated with 2yDFS in the 53 patients (Table 1). The size of the alteration associated with 2yDFS ranged from a single BAC (loss of chr17, at 16.3 Mb) to almost the entire chromosome arm (loss of chr4q, 60.9 Mb-190.6 Mb). The alterations were present in a minimum of 5 patients (range, 5–30 patients, median 14.5). All regions were associated with poor patient outcome with the exception of gain of a region on 12q (82.3–83.6 Mb), which was associated with good patient outcome (Odds Ratio, OR = 0.18). There were four regions that had an infinite OR, as all patients who had the alteration had poor outcome (gain chr9, 72.7–120.8 Mb; gain chr18, 0.1–4 Mb; loss chr12, 41.2–47.4; loss chr17, 16.3 Mb)

**Table 1:** Genomic regions and putative target genes associated with 2yDFS in NSGCT patients.

**Chr**	Region (in Mb)	No.	Alt.	*P* Value	OR	95% C.I.	Sig Gene	FDR (%)	Target Genes with >2X exp Rel to Samples w/o Alteration	Target Genes with >3X exp Rel to Normal Samples
9	72.7–120.8	7	gain	0.017	INF	N/A	94	9.92	*KLF4*, *PCSK5*, *SMC2L1*, *UBQLN1*	*TXN*
12	82.3–83.6	14	gain	0.041	0.18	0.04–0.80	none	N/A	N/A	N/A
14	62.9–97.5	10	gain	0.037	10.9	1.14–103.98	152	9.84	*211429_s_at*, *ALDH6A1*, *MED6*, *NRXN3*, *PAPOLA*, *PGF*, *SGPP1*	*211429_s_at*, *ACTN1*, *DLST*, *NRXN3*, *PGF*
18	0.1–4	5	gain	0.036	INF	N/A	19	6.86	*HSRTSBETA*, *KIAA0650*, *LPIN2*, *TGIF*, *TYMS*, *YES1*	*TGIF*
20	23.5–26.1	16	gain	0.025	5.20	1.32–20.54	11	7.27	*C20orf56*, *CST1*	*C20orf56*, *CST1*, *FOXA2*
1	18.0–28.7	15	loss	0.011	10.21	1.75–59.65	none	N/A	N/A	N/A
1	95.4–101.5	14	loss	0.046	4.89	1.05–22.84	none	N/A	N/A	N/A
4	1.1–38.1	30	loss	0.020	4.50	1.31–15.42	17	8.93	none	*CPEB2*, *FBXL5*, *FLJ90575*
4	60.9–190.6	28	loss	0.003	7.92	2.12–29.60	2	0	*SYNPO2*	*SYNPO2*
6	0.1	17	loss	0.043	5.50	1.22–24.81	2	0	none	*DUSP22*
9	0.2–35.4	23	loss	0.013	6.25	1.58–24.80	none	N/A	N/A	N/A
9	123.3–128.5	26	loss	0.017	4.86	1.37–17.19	none	N/A	N/A	N/A
12	41.2–47.4	7	loss	0.026	INF	N/A	5	0	*NELL2*, *SLC38A1*	none
17	16.3	7	loss	0.046	INF	N/A	1	0	none	*UBB*

Chr: chromosomal location of copy number alteration associated with outcome

Region: chromosomal region associated with outcome

No.: number of tumors that display the copy number alteration

Alt.: copy number alteration type (gain or loss)

p-value: p-value of the association between copy number alteration and outcome

OR: odds ratio for the event

95% C.I.: 95% confidence interval of the odds ratio

Sig. Gene: number of significantly differentially expressed genes that map to region

FDR: false discovery rate of significant genes mapping to region

To determine which, if any, genes were potential targets of these alterations, we first identified genes that mapped to the altered regions, and then used SAM to identify significantly differentially expressed genes as described in the methods. In particular, we focused on genes that showed at least a two-fold difference in expression, since smaller magnitude changes would likely be difficult to assess in clinical specimens. For nine of the regions, we were able to identify significant genes that were differentially expressed between tumors with alterations and tumors with normal copy number and/or normal testes specimens (Table 1). In total, there were 39 probe sets (representing 31 unique genes) that were differentially expressed at greater than a two-fold level between tumors with alterations and the tumors without alterations at the corresponding region.

### 5yDSS Associations

For 5yDSS, we identified 16 regions that showed associations with poor patient outcome, which were present in a minimum of 3 patients (range, 3–30 patients, median 16.5; Table 2). The smallest region was ~5 Mb in size (chr17, 0.7–5.9 Mb), while almost all of chr4 losses (0–58.2 Mb and 72.7–90.6) showed associations with outcome. Two regions had infinite OR, as a result of all patients with the alteration dying of disease (gain of chr18, 26.1–77.6 Mb; loss of chr2, 78.5–118.7 Mb). Not surprisingly, there were several regions associated with 5yDSS that showed full or partial overlap with 2yDFS outcome-associated regions, including gain of chr14 (62.9–76.5 Mb), and losses of chr1 (19.2–26.4 Mb), chr4 (1.1–38.1 Mb and 72.7–190.6 Mb), and chr9 (0.2–35.9 Mb and 123.3–128.5 Mb).

**Table 2:** Genomic regions and putative target genes associated with 5yDSS in NSGCT patients.

Chr	Region (in Mb)	No.	Alt	*P* Value	OR	95% C.I.	Sig Genes	FDR (%)	Target Genes with >2X exp Rel to Samples w/o Alteration	Target Genes with >3X exp Rel to Normal Samples
14	38.7–41.8	8	Gain	0.026	9.00	1.32–61.14	6	10	none	none
14	62.9–76.5	11	Gain	0.018	7.93	1.48–42.58	104	9.76	*ALDH6A1*, *MED6*, *SGPP1*	*ACTN1*, *DLST*, *PGF*
18	26.1–77.6	3	Gain	0.029	INF	N/A	74	9.34	*226974_at*, *227542_at*, *233446_at*, *239911_at*, *ACAA2*, *B4GALT6*, *C18orf10*, *DSC2*, *DSG2*, *FLJ20793*, *KIAA1468*, *LOC284267*, *ME2*, *MYO5B*, *NARS*, *NEDD4L*, *P15RS*, *POLI*, *RKHD2*, *SLC39A6*, *SMAD4*, *TNFRSF11A*	*226974_at*, *227542_at*, *233446_at*, *239911_at*, *ACAA2*, *DSC2*, *DSG2*, *MYO5B*, *NEDD4L*, *TNFRSF11A*
1	19.2–26.4	15	Loss	0.040	5.04	1.13–22.50	None	N/A	N/A	N/A
1	83.8–92.1	13	Loss	0.034	5.63	1.24–25.49	None	N/A	N/A	N/A
2	78.5–118.7	5	Loss	0.013	INF	N/A	114	9.69	*238768_at*, *ACTR3*, *ANAPC1*, *ASCC3L1*, *EIF2AK3*, *INPP4A*, *LOC129531*, *MAP4K4*, *RANBP2*, *RW1*, *SEPT10*, *STARD7*, *TGOLN2*, *TXNDC9*	*241234_at*, *BUB1*, *C2orf23*, *GCC2*, *JMJD1A*, *LIPT1*, *MAP4K4*, *RANBP2*, *RANBP2L1*, *SEPT10*, *UXS1*, *VPS24*
2	163.1–178.6	10	Loss	0.026	7.27	1.31–40.43	29	7.78	none	*AGPS*, *LOC401022*, *MTX2*
4	0–58.2	30	Loss	0.009	5.82	1.53–22.17	14	5.42	*HOP*	*CPEB2*, *FBXL5*, *HOP*, *KIAA1458*
4	72.7–190.6	28	Loss	0.001	10.70	2.46–46.53	2	0	*SYNPO2*	*SYNPO2*
6	78.6–84.6	19	Loss	0.027	4.71	1.25–17.71	None	N/A	N/A	N/A
7	102.6–121.2	6	Loss	0.015	17.50	1.56–196.33	None	N/A	N/A	N/A
9	0.2–35.9	23	Loss	0.002	8.40	2.12–33.29	None	N/A	N/A	N/A
9	116.9–128.5	26	Loss	0.031	4.67	1.25–17.36	None	N/A	N/A	N/A
14	38.7–41.8	19	Loss	0.024	5.14	1.29–20.52	None	N/A	N/A	N/A
14	62.9–70.5	18	Loss	0.047	4.37	1.07–17.79	None	N/A	N/A	N/A
17	0.7–5.9	12	Loss	0.025	7.20	1.35–38.33	44	7.06	*CXCL16*, *SERPNIF1*	*GPS2*, *SERPINF1*

Chr: chromosomal location of copy number alteration associated with outcome

Region: chromosomal region associated with outcome

No.: number of tumors that display the copy number alteration

Alt.: copy number alteration type (gain or loss)

p-value: p-value of the association between copy number alteration and outcome

OR: odds ratio for the event

95% C.I.: 95% confidence interval of the odds ratio

Sig. Gene: number of significantly differentially expressed genes that map to region

FDR: false discovery rate of significant genes mapping to region

For eight of the regions, we were unable to identify any genes that showed significant differential expression between tumors with the alteration compared to those with normal copy number. For one other region (gain of chr14, 38.7–41.8 Mb), we identified 10 significant probe sets, but none showed a two- or three-fold difference relative to tumors with normal copy number or normal testes specimens respectively. From the remaining seven regions, we identified 75 probe sets (representing 62 unique genes) that were significantly differentially expressed between tumors with the alterations and those with normal copy number (Table 2). As expected, several of the genes showed overlap with the genes associated with 2yDFS, including *ACTN1*, *ALDH6A1*, *CPEB2*, *DLST*, *FBXL5*, *MED6*, *PGF*, *SGPP1*, and *SYNPO2*.

### Validation of Copy Number Changes

Although we have previously published the copy number data, we felt it was important to validate the copy number calls in several samples since we were now associating the alterations with outcome. We confirmed the copy number changes in three tumor samples using the Agilent 44K chip platform for copy number changes. There was excellent agreement in the identification of altered genomic regions between the different platforms, with concordance rates of 92.7, 97.6, and 97.2, confirming the accuracy of the copy number alteration calls (S1 Fig). The processed copy number data from the Agilent arrays is available as S2 Table.

### Outcome Prediction

We used the tumors that we had identified the differentially expressed genes to build predictive models for both 2yDFS and 5yDSS, and then tested the models in an independent validation set of 54 NSGCT specimens (49 NSGCT for 5yDSS) for which we had expression but not genomic data, to avoid over-fitting of the predictive model.

For 2yDFS, the full set of 39 probe sets was optimal, but only gave a prediction rate of 64.2% in the independent tumor set. This is consistent with our findings from our expression only studies [5], in which we were unable to identify a strong signature that predicted 2yDFS.

For 5yDSS, the full set of 75 probe sets was optimal, giving a prediction rate of 75.6% in the independent data set. Since 2yDFS is also strongly correlated with 5yDSS (i.e, patients with poor 2yDFS are more likely to have poor 5yDSS), and given the high degree of overlap between the regions and genes identified for the two outcome endpoints, we decided to model the effect of including the non-overlapping genes identified in the 2yDFS screen (101 total probe sets) with the 5yDSS gene set. We built a model with the combined gene sets in the training cohort and tested it in the independent validation cohort. Interestingly, the combined set of genes gave a superior performance in the independent data set (prediction rate of 79.6%). This result was not unexpected, since patients with poor 2yDFS would be more likely to have poor 5yDSS, as mentioned above.

**Fig 1:**
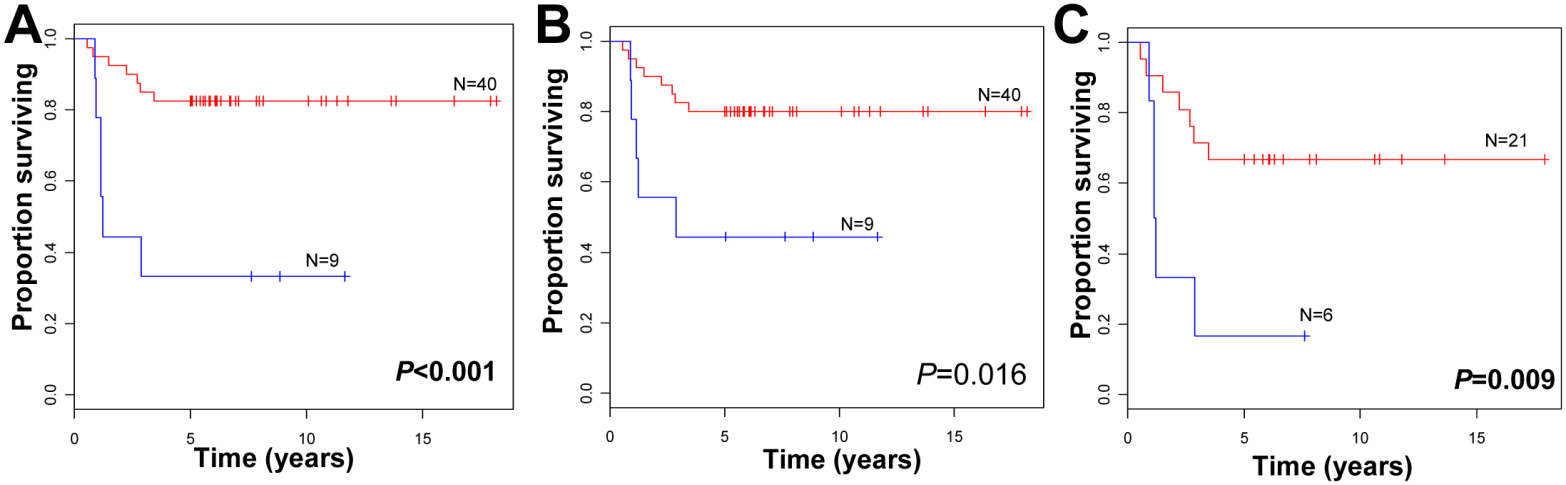
**A.** Kaplan-Meier curves show differential survival of patients with predicted good (red) and poor (blue) outcome using the full set of genes associated with 2yDFS and 5yOS. **B.** Kaplan-Meier curves show differential survival of patients with predicted good (red) and poor (blue) outcome using genes associated with 5yOS. **C.** Kaplan-Meier curves show differential survival of patients with intermediate and poor IGCCCG risk with predicted good (red) or poor (blue) outcome using genes associated with 2yDFS and 5yOS.

Based on the predicted outcome using only the 5yDSS associated probe sets or the combined set of probe sets in the independent tumor set, we performed survival analysis by the Kaplan-Meier method (Fig 1A and 1B). In both cases, patients with good predicted outcome had significantly better survival than those with poor predicted outcome (*p*<0.001 when using all genes; *p* = 0.018 when using the genes associated with 5yDSS). For the full set of genes, a multivariate analysis using linear regression and IGCCCG classification as a continuous variable showed that the gene set was an independent prognostic factor (*p* = 0.017 for gene model, *p* = 0.002 for IGCCCG risk). Similarly, if the IGCCCG was used as a binary variable (with intermediate and poor risk classes combined to account for the relatively small sample size; N = 27), the gene set was still significant (*p* = 0.016 for gene model, *p* = 0.012 for binary IGCCCG risk). As expected, when we applied the gene predictor to patients with elevated risk (i.e., intermediate or poor risk), there was a significant difference in patient survival, as shown by the Kaplan-Meier method (Fig 1C), indicating that the gene signature may have clinical utility in stratifying high risk patients. Anecdotally, there was only one good risk patient in the independent set of tumors that died of disease, and this patient was predicted to die from disease by the combined gene model.

**Fig 2:**
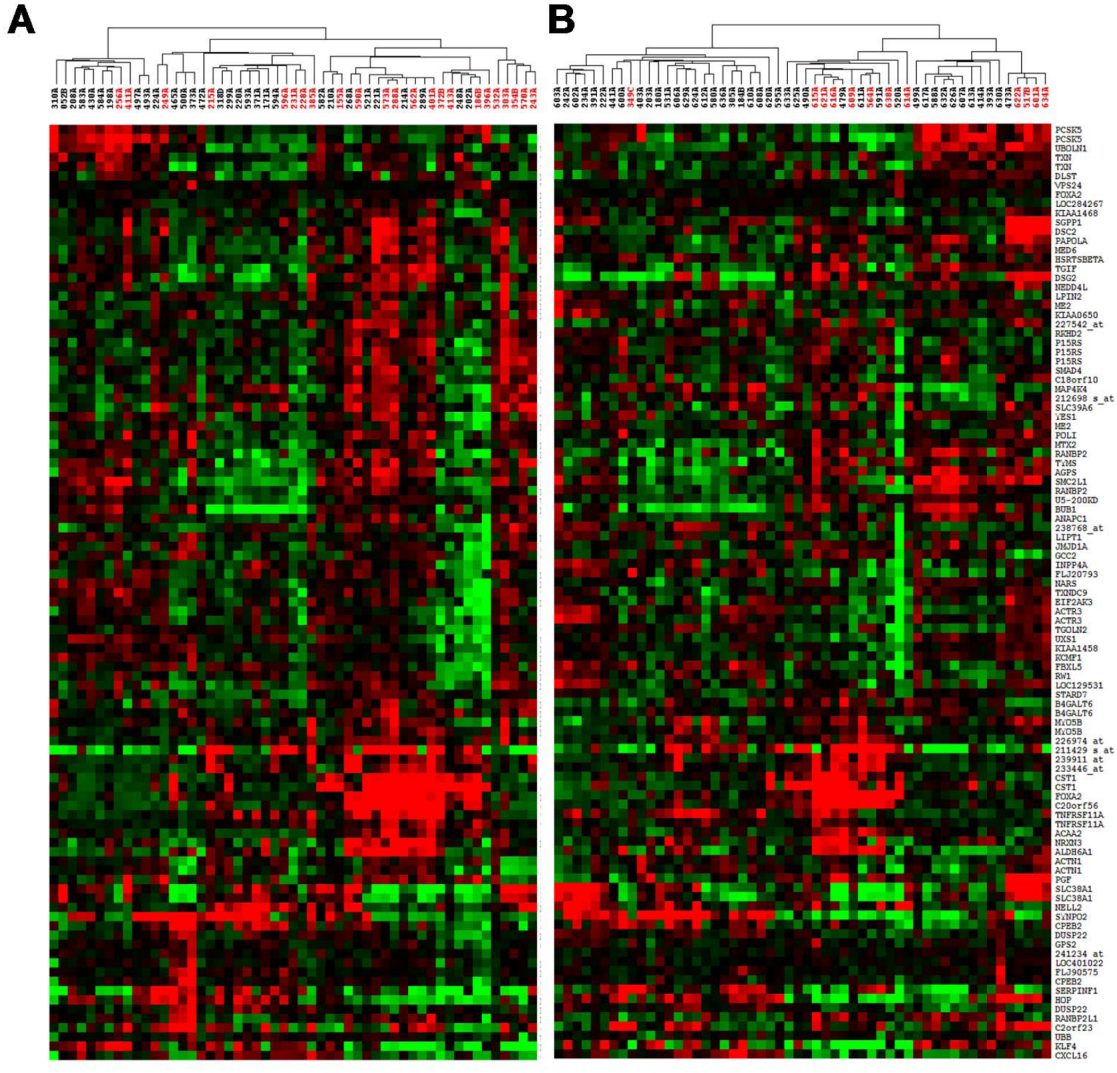
Hierarchical clustering of outcome associated genes in training set (A) and validation set (B) showing poor outcome in distinct subsets of tumors. Red samples indicate patients who died from disease prior to 5 years.

Since the approach of identifying outcome-associated genes using this combined genomic and expression profiling would be expected to identify genes that were expressed in subsets of patients, we performed hierarchical clustering on both sets of tumors (i.e. combined array CGH-expression set and the independent expression set) to visualize the tumor and gene grouping patterns. As expected, there were mixtures of small, distinct subgroups consisting of good and poor outcome patients based on variable gene expression across these specimens (see Fig 2), as opposed to our previous expression-only based study which showed strong separation of good and poor outcome patients into distinct groups [5].

### Comparison to expression gene sets

We compared the gene lists that we generated in the present study to the ones from our expression only study [5]. Of the 104 unique probe sets from the combined CGH-expression study and the 174 probe sets from the expression only study, there were three genes that showed overlap (*SYNPO2*, *HOP*, and *CPEB2*). Interestingly, all three of these genes mapped to chr4, which was lost in a high percentage of cases.

## Discussion

Development of high throughput screening techniques over the past decade have led to the identification of a number of gene sets that are predictive of patient outcome in a number of different cancers, including breast [14], lung [15], and prostate [16]. Due to the relative rarity of GCTs, there have not been any large scale studies to identify such predictive gene sets in testes cancer until recently. We completed a large study with both training and validation sets to identify genes associated with patient outcome using an expression based approach [5]. While these gene sets are useful, one limitation of our previous approach is that gene expression is averaged over the entire group. As a result, genes that are only expressed in a small subset of patients but are highly correlated with patient outcome may be missed. While computational approaches may allow the identification of such changes [6], we chose a different approach to identifying genes that are found only in subsets of tumors. We used array CGH, a well-accepted and accurate method for measuring copy number alterations [17], to identify genomic regions associated with patient outcome. We then identified significant genes from our expression profiling studies that mapped to those regions. We were then able to build an outcome prediction model and validate it in an independent set of tumor specimens.

This approach allowed us to identify altered genomic regions that were associated with both 2yDFS as well as 5yDSS. We identified a number of regions that overlapped between the two endpoints, which would be expected since patients that had poor 2yDFS (i.e., they had an incomplete response to chemotherapy or relapsed within two years) would be more likely to have poor 5yDSS than patients who had a complete response. These regions included gain of chr14 and losses on chr1, 4, and 9.

Additionally, we hypothesized that some of the regions we identified might also be associated with cisplatin resistance *in vitro*, since poor patient outcome is strongly correlated with cisplatin resistance. However, none of the regions that previously have been associated with cisplatin resistance in GCT cell lines such as 6q26-27, 10p14, or 16q22-23 [18, 19] were identified in the present study. This could be an issue with sample size, as the cell line studies were limited to a small number of samples, or alternatively, could reflect differences between *in vitro* and *in vivo* specimens. Similarly, a previous study from our lab identified genomic amplifications that were associated with poor patient outcome in tumor specimens. One region on 20q (30.6–31.4 Mb) was of borderline significance in the present study (*p* = 0.06) and corresponded to a region that we previously showed was a site of chromosomal amplification associated with poor patient outcome [20]. However, none of the other regions identified in the previous study were associated with outcome in our present study, although this could be due to the fact that we examined only copy number changes as opposed to high level amplifications. Finally, we had also previously identified loss of 12q as the site of a potential tumor suppressor gene in GCTs [21]. We identified loss of 12q12 (41.2–47.4 Mb) as being associated with poor patient outcome, which corresponds to a region of loss of heterozygosity that we previously found in 27% of the tumors that we studied. Within this genomic region, we identified two genes that showed significant changes in expression in tumors that had the loss (*NELL2* and *SLC38A1*). Interestingly, a recent study showed that *NELL2* is a target gene for *HNF4*α, which is frequently lost in kidney cancer, and that expression of *NELL2* contributes to *HNF4*α mediated inhibition of proliferation in kidney cells [22].

Given that we did not have an additional CGH cohort available for validation, we instead focused on genes that mapped within the regions of interest. Since we knew the types of changes (i.e., gain or loss), we used SAM to identify genes that showed at least 2 fold expression level changes in the expected direction (increase for gains, decrease for losses) in order to maximize their potential predictive power. This was in part due to the fact that any assays that are developed in the future will likely require at least 2-fold changes in expression in order to be reproducibly detected between good and poor outcome patient groups.

The genes we identified showed very little overlap with the genes that we previously identified in our expression only approach [5]. This was not surprising, since the combined aCGH-expression approach would be expected to identify genomic regions and genes that had alterations associated with outcome that were present in subsets of samples, whereas the expression only approach identifies genes that on average, are expressed in either good or poor outcome groups. The exception to this was the presence of three genes (*SYNPO2*, *HOP*, and *CPEB2*) in both studies, which was not unexpected since all three map to chr4, which is lost in a large proportion of tumors, and thus could be expected to be present in a large enough number of samples to be identified without genomic information. Interestingly, both *HOP* and *SYNPO2* have been implicated as tumor suppressor genes, *HOP* in female choriocarcinomas associated with hydatiform moles and esophageal tumors, and *SYNPO2* in bladder and prostate cancer [23, 24].

The performance of combined CGH-expression classifier is difficult to compare with the predictive ability of our previously expression only based classifier [21] since the validation sets differed in composition. This was due to the fact that some of the samples in the original training set did not have array CGH data available, while some of the original validation set did have array CGH data available, leading to different makeup of both the training and validation sets between the two studies. Unfortunately, no independent data sets exist to our knowledge, and thus a rigorous comparison of the predictive abilities of the two methods is not possible at this time. As a result, at this time we can only state that the performance of our new predictor appears similar to that of our original expression predictor; although the gene sets share almost no commonality. Anecdotally, we did observe an individual case that differed between the two sets. This was sample 616A, from a patient who died of disease, who was predicted to have poor outcome by the expression-based predictor, but good outcome by the combined CGH-expression-based predictor. Interestingly, all other misclassified cases that overlapped between the two validation sets were consistent: one case in which the patient died of disease (634A), in which both predictors classified the patient as a good outcome case, and two cases in which the predictors gave a poor prognosis but the patients survived past the 5y cutoff (611A, 626A). These latter cases may represent surgical cures, which cannot be predicted by gene expression methods.

Interestingly, for many of the regions that were associated with outcome, we were unable to identify significantly differentially expressed genes mapping to those regions. There may be several explanations for this. First, it is possible that methylation events may lead to silencing of the target genes in poor outcome patients without the genomic alteration, and thus would be difficult to detect as being differentially expressed when performing the comparison. Another possibility is that small non-coding RNA’s could map to these regions, and their expression could influence expression of other vital target genes that lie outside the altered regions. Indeed, microRNAs that target TP53 have been identified in GCT cell lines [25].

There are several minor potential problems that will need to be addressed in future studies. First is the small sample size of our validation cohort, particularly those with poor outcome (N = 10). Although statistically valid, we plan to perform more extensive validation in larger cohorts in future studies. Second, we ideally would have used matched normal samples as the copy number control to eliminate any small polymorphic regions that might have been present in patient samples that might have been identified as associated with outcome. Unfortunately, matched normal specimens were not readily available to us for this study. However, we feel it is unlikely that any of the genomic changes associated with outcome are due to germline polymorphisms, since most of the regions we identified are much larger than typical polymorphisms that have been observed in individuals.

The genes that we identified were highly predictive of patient outcome in an independent set of tumor specimens, and represent a non-overlapping set of genes from the predictive gene sets that we had previously identified. As with our other predictive gene set [5], this gene list is an independent prognostic indicator when combined with IGCCCG risk stratification. Additionally, the gene set appears to be effective in further stratifying patients who are at higher risk, which could be beneficial in identifying patients who would benefit from more stringent monitoring and/or aggressive chemotherapy. Adaptation of these genomic regions or gene sets into clinically applicable assays could improve outcome prediction in GCT patients, and may provide novel targets for therapeutic intervention.

## Supporting Information

S1 Fig Comparison of copy number alteration calls made between array CGH (black) and Agilent 44K snp chips (red) for three GCT samples. The y-axis in each plot denotes copy number level (either gain, normal, or loss) and the x-axis denotes position, ordered by chromosome from 1 to X. Note the excellent concordance scores for the copy number calls, and the gain of 12p in all three samples.
(TIF)

S1 Table Table outlining clinical features for the patients included in this study. (XLSX)

S2 Table Copy number calls for the three tumors run on the Agilent snp chips, ordered from 1 to X chromosome. (XLSX)
