# Supplementary figures and images for: Development and Validation of a Gene-Based Model for Outcome Prediction in Germ Cell Tumors Using a Combined Genomic and Expression Profiling Approach
Source: PLoS One. 2015 Dec 1;10(12):e0142846. doi: 10.1371/journal.pone.0142846 (PMC4666461; doi:10.1371/journal.pone.0142846)

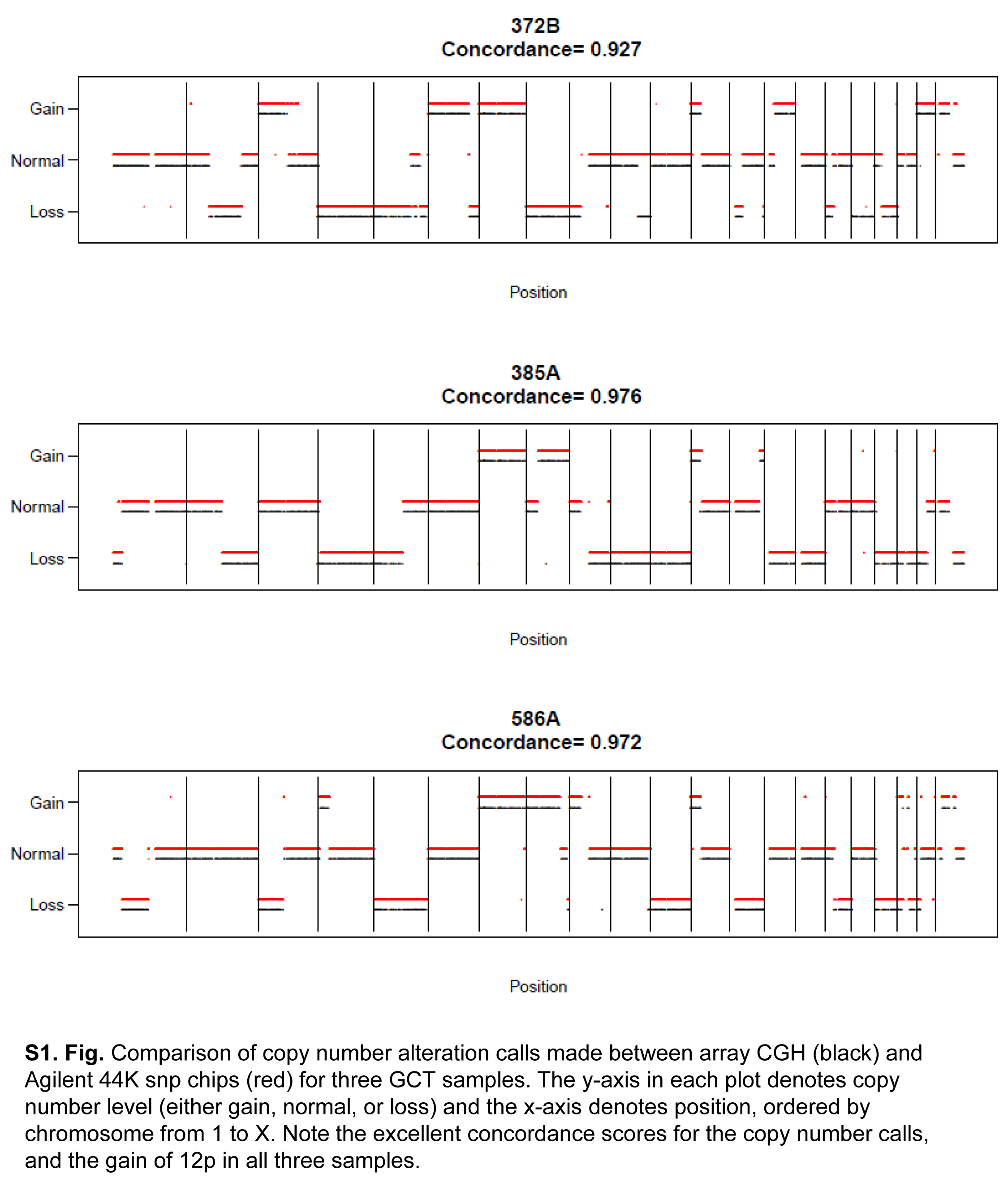

Supplement: S1 Fig — The y-axis in each plot denotes copy number level (either gain, normal, or loss) and the x-axis denotes position, ordered by chromosome from 1 to X. Note the excellent concordance scores for the copy number calls, and the gain of 12p in all three samples. (TIF) [file pone.0142846.s001.tif]
